# Research on the U-shaped relationship between marketization process and corporate capital structure: Evidence from Chinese listed companies

**DOI:** 10.1371/journal.pone.0331367

**Published:** 2025-09-02

**Authors:** Junzhou Xu, Ling Zhang

**Affiliations:** 1 Department of Economics and Management, Hubei Polytechnic University, Huangshi, China; 2 Key Research Institute of Humanities and Social Sciences of Hubei Province “The Digital Economy Industrialization Development Research Center of Eastern Hubei”, Huangshi, China; 3 School of Software and Internet of Things Engineering, Jiangxi University of Finance and Economics, Nanchang, China; Universiti Malaysia Sabah, MALAYSIA

## Abstract

This paper explores the U-shaped relationship between marketization and corporate capital structure using panel data from Chinese listed firms (2012–2021). Applying fixed effects and nonlinear regressions, we find that as marketization increases, leverage first declines and then rises, confirming a robust U-shaped pattern. In early reforms, improved institutions reduce debt reliance; in advanced stages, enhanced capital markets facilitate debt access. Additionally, state-owned equity weakens this relationship, while fixed asset proportion strengthens it. The findings enrich capital structure theory under institutional transition and offer policy and strategic insights for emerging markets.

## Introduction

Market-oriented reforms have played a crucial role in driving China’s economic growth. Marketization, as a systemic transition from planned to market economies, entails not just simple changes in regulations but a series of transformations in economic, social, and legal institutions—a substantial institutional shift [1]. Marketization is a systematic reform involving the transformation of government-market relations, which has far-reaching institutional impacts [2]. Adapting to the development of marketization is a significant challenge faced by every enterprise.

The capital structure of a business refers, on one hand, to the ratio of liabilities to total assets and the structure of liabilities and, on the other hand, to the shareholder structure of the owner’s equity. Capital structure is one of the core variables of a company’s long-term value and agency mechanism [3]. Capital structure involves the specific combination of long-term debt and equity financing for a company [4]. Modern financial theory suggests that the choice of capital structure is crucial as it not only influences the market value of a company but is also associated with corporate governance and macroeconomic operations.

Research in the field of capital structure requires identifying the key factors that drive corporate financing decisions and capital structure [5]. After nearly 60 years of efforts by numerous scholars, several significant factors influencing corporate capital structure decisions have been identified. These include stakeholders [6], market competition [7], import and export product markets [8], dynamic adjustments [9], macroeconomic factors, among others. Graham and Leary (2011) decomposed the variance of capital structure into inter-industry differences, intra-industry differences, and within-firm differences over time, using empirical variables derived from capital structure theories to examine their explanatory power. The results revealed that the core explanatory variables developed by current capital structure theories have limited explanatory power [10] In particular, it is not good at explaining the dynamic changes in capital structure among companies [11].

To deepen understanding of how institutional environments influence capital structure decisions, prior studies have explored both developed and emerging markets. Booth et al. (2001) found that while traditional determinants retain some relevance in developing countries, country-specific institutional factors are crucial [12]. Delcoure (2007) observed modified pecking order financing behaviors in Central and Eastern European transitional economies, influenced by variations in legal systems, banking sector development, and investor protections [13]. Similarly, De Jong et al. (2008) highlighted the interplay between firm-level and country-level institutional factors globally. These international perspectives underscore the importance of local institutional environments and justify the focus on China’s unique marketization trajectory [14].

However, a relatively underexplored area is how institutional transitions—specifically, the process of marketization—affect corporate capital structure in emerging economies such as China. Although some research has examined macroeconomic reforms’ impact on firm behavior, few have systematically analyzed the potential nonlinear effects of marketization on leverage decisions.

This paper addresses this gap by investigating the U-shaped relationship between marketization and corporate capital structure. It contributes to the literature in two ways: first, by providing empirical evidence on how institutional changes influence financing behavior during economic transition; second, by exploring the moderating effects of state ownership and fixed asset intensity on this relationship. These contributions offer new insights for capital structure theory and institutional economics in emerging market contexts.

Specifically, this study seeks to answer the following research questions:

1. Does the degree of marketization influence Chinese firms’ capital structure decisions in a nonlinear manner?
2. How does state ownership moderate the relationship between marketization and corporate capital structure?
3. What role does fixed asset intensity play in moderating marketization’s effects on firm leverage?

The remainder of this paper is organized as follows: Section 2 develops the theoretical framework and hypotheses; Section 3 describes data sources, variable definitions, and empirical methodology; Section 4 presents the empirical results, moderation analyses, and robustness checks; and Section 5 concludes with main findings and policy implications.

## Section 2: Literature review and hypotheses

The marketization process represents a systemic reform in China’s transition from a planned to a market economy. It involves a series of changes not only in regulatory systems but also in economic, social, legal, and even political institutions [15]. We utilize the Provincial Marketization Process Index compiled by the China National Economic Research Institute to examine the impact of the marketization process on corporate capital structure. The components of the marketization process include the relationship between the government and the market, the degree of development of the non-state-owned economy, the level of development in product markets, the level of development in factor markets, the level of market development, and intermediary organizations and legal environments [15].

The elements constituting the marketization process have implications for corporate capital structure: (1) Government intervention and policy measures affect corporate financing channels and costs, subsequently influencing the composition of capital structure. The role of government profoundly shapes the evolution of capital structure [3,16]. Government intervention can also be realized through increased debt, particularly in the capital restructuring of state-owned enterprises [17]. Government intervention can also be realized through increased debt, particularly in the capital restructuring of state-owned enterprises [18]. (2) Government pressures and policy orientations may impact corporate financing needs and methods, thereby influencing capital structure. Local governments may exert pressure on companies to meet specific standards [19]. This aligns with state-directed reforms that disproportionately impact non-state enterprises in competitive sectors and low-marketization regions, as shown in [20]. (3) Under the impact of import competition, companies may face a contraction in internal financing sources, leading them to prefer external financing such as debt to meet their funding needs, thereby affecting corporate capital structure. Companies affected by Chinese import competition choose external financing as a rational choice due to the shrinkage of internal financing sources [21]. (4) The expansion of the labor market may have a negative impact on corporate capital structure, especially for labor-intensive enterprises and industries with lower financing constraints. The labor market scale has a significant negative impact on corporate capital structure, particularly in companies with lower financing constraints and labor-intensive industries [22]. (5) The increase in labor protection levels can significantly reduce corporate debt, and the higher the law enforcement efficiency in the company’s region, the more this negative correlation will appear in non-state-owned enterprises [23]. This finding is reinforced by evidence from labor standardization reforms that differentially impact non-state firms in low-marketized regions [20]. The literature cited above illustrates the significant relevance of examining the impact of the marketization process on corporate capital structure.

In the early stages of marketization, firms often operate within underdeveloped financial systems characterized by limited financing channels, high transaction costs, and weak investor protection mechanisms. Under such circumstances, internal financing becomes the preferred option, as it helps reduce capital costs and avoid the risks associated with information asymmetry in external financing. This behavior aligns with the core tenets of Pecking Order Theory, which suggests that due to asymmetric information, firms follow a financing hierarchy: they prefer retained earnings first, followed by debt, and only issue equity as a last resort [16]. Therefore, as marketization progresses from a low to moderate level, firms gain better access to external debt markets and face lower financing costs, prompting a gradual shift from internal to external debt financing—resulting in a rise in leverage.

However, as marketization continues to deepen and capital markets become more efficient and liquid, the logic behind firms’ financing decisions shifts. In highly developed markets, capital becomes more accessible and equity valuations more sensitive to market perceptions. Managers are thus able to exploit favorable stock valuations by issuing equity when they perceive their firm to be overvalued, in order to reduce overall capital costs and avoid the financial risks associated with excessive debt. This behavior is consistent with Market Timing Theory, which posits that firms prefer equity financing during periods of high valuation and avoid it when their stock is undervalued [24]. Consequently, even though debt financing remains available, firms increasingly substitute equity for debt, leading to a decline in leverage. This reversal in the leverage trend forms the declining segment of the U-shaped curve.

In summary, the relationship between marketization and corporate capital structure is not linear, but rather shaped by the interplay of multiple theoretical mechanisms. In the early and middle stages, financing decisions are primarily driven by the logic of Pecking Order Theory, where financial frictions and information asymmetry dominate. In the advanced stages of marketization, however, firms are guided more by market timing considerations and strategic behavior based on equity valuation. The changing dominance of these financing logics across different stages of market development ultimately gives rise to a non-linear, U-shaped relationship between marketization and firm leverage.

Notably, similar patterns have been observed in other transitional economies. For instance, Delcoure (2007) found that firms in Central and Eastern European countries follow a modified pecking order logic, with leverage decisions influenced by institutional constraints, legal environments, and the development of financial markets [13]. Booth et al. (2001) showed that although traditional capital structure determinants are partly applicable in developing countries, institutional differences create persistent cross-country heterogeneity [12]. De Jong et al. (2008) further highlighted that firm-level and country-level determinants interact differently across economies, suggesting that institutional context significantly shapes capital structure behavior [14]. These findings reinforce the view that non-linear capital structure dynamics under institutional transitions, such as a U-shaped pattern, are not unique to China but may reflect a broader phenomenon in emerging and transitioning economies.

Based on this theoretical framework, the following hypothesis is proposed:

H1: There is a U-shaped relationship between the marketization process and corporate capital structure.

Differences in the ownership nature of listed companies will significantly influence the distribution of leverage ratio reasonable thresholds [25]. State-owned enterprises obtain policy-based financing support through their relationship with the government and are therefore more inclined to operate with high leverage, State-owned enterprises tend to have a high leverage structure due to the externalization of agency costs [3]. State-owned equity-holding companies benefit from close connections with the government and can obtain more policy loans or government intervention loans compared to non-state-owned equity-holding companies, leading to lower financing costs through banking channels and, thus, having the motivation to increase leverage.

Two-stage typical case analyses. First, during the period from 2012 to 2017, the financial leverage ratio of Chinese enterprises reached a peak in 2013. In November 2008, China proposed the “Four Trillion” economic stimulus policy to stimulate economic development directly. As investment projects were transformed, corporate profitability increased, and the ability of companies to repay debt improved, the leverage ratio of enterprises decreased year by year after 2013. State-owned equity-holding companies, backed by the government, could obtain more loans, further motivating their development and hindering the decline in leverage. For example, Anhui Conch, with the Anhui Provincial State-owned Assets Supervision and Administration Commission, holding 35.07% of shares, had asset-to-liability ratios of 29.63%, 27.4%, and 26.72% in 2013–2015, respectively.

Second, during the period from 2017 to 2021, after the Chinese government proposed the supply-side reform, the level of marketization increased, the market became fairer, and competition intensified, directly leading to an increase in corporate financial leverage. In August 2020, the People’s Bank of China and the China Banking and Insurance Regulatory Commission proposed the “three red lines” policy for real estate companies, including an asset-to-liability ratio not exceeding 70% after deducting pre-received funds, a net debt ratio not exceeding 100%, and a cash short-term debt ratio greater than 1. After the policy was introduced, state-owned equity-holding companies in the real estate industry played a leading role, hindering the increase in corporate leverage. For example, China Overseas Development and Investment, with 51.33% held by China State Construction Group, had asset-to-liability ratios of 60.14%, 58.95%, and 59.15% in 2020–2022, respectively.

Based on this, the following hypotheses are proposed:

H2: State ownership has a negative moderating effect on the relationship between the marketization process and corporate capital structure.

The proportion of fixed assets refers to the proportion of fixed assets to the total assets of an enterprise. In a company’s financing decisions, a high proportion of fixed assets can affect capital structure. Our research evidence for 295 mining and manufacturing companies strongly indicates that unique company-specific assets and skills are the most critical determinants of capital structure to date [26]. Using extensive sample data from US-listed companies, there is a positive correlation between asset liquidity and secured debt, while there is a curvilinear relationship between asset liquidity and unsecured debt [27]. For unsecured debt, greater liquidity increases the credit spread of corporate debt and reduces the optimal leverage ratio [28].

The increase in the proportion of fixed assets improves the company’s mortgage capacity, thereby reducing external financing barriers and increasing the debt level.The mortgageability of corporate assets is positively correlated with capital structure [11,27]. In the early stages of the marketization process, the proportion of fixed assets in enterprises decreases, often seen as the liquidation of fixed assets, leading to cash flow for enterprises. Companies weigh market opportunities and the cost of funds. If market opportunities are not favorable, they usually choose debt financing, resulting in a decrease in the leverage ratio of enterprises. In the mid-to-late stages of the marketization process, competition among enterprises intensifies, and companies need to gain more competitive advantages to maintain their positions. For most industries, scale is an important way to reduce costs, especially in extensive industries that add a large number of fixed assets, expand capacity, establish more production lines, increase fixed assets, increase corporate debt, and increase corporate leverage.

Two-stage typical case analyses. First, during the period from 2012 to 2017, companies had just experienced the impact of the 2008 US financial crisis. To increase cash flow, some fixed assets were disposed of, leading to a decrease in the proportion of fixed assets. When sold fixed assets were converted into cash income, the company could use it for debt financing, reducing the leverage ratio of the company. Second, during the period from 2017 to 2021, for example, in 2020, the national government introduced a 3 trillion yuan stimulus policy. Observing that construction companies purchased a large number of mechanical equipment directly led to an increase in fixed assets and an increase in corporate leverage. For example, Sany Heavy Industry, with asset-to-liability ratios of 49.72%, 53.91%, and 53.02% in 2019–2021, respectively. This progression mirrors how structural industrial reforms—prioritizing scale, capital deepening, and fixed asset expansion—are embedded in China’s gradualist market reform strategy [29].

Based on this, the following hypothesis is proposed:

H3: The proportion of fixed assets has a positive moderating effect on the relationship between the marketization process and corporate capital structure.

## Section 3: Data and methodology

### Data

The data used in this study are sourced from listed companies on the Shanghai Stock Exchange and Shenzhen Stock Exchange from 2012 to 2021. The variable for the marketization process is derived from the “China Marketization Index - Industry-specific Marketization Relative Progress Report,” while other data are obtained from the WIND database and CSMAR database. We applied the following criteria to filter the sample: (1) Exclude financial listed companies; (2) Exclude samples with negative net assets; (3) Exclude samples with missing relevant data; (4) Trim the sample at the 1% and 99% levels. In the end, we obtained panel data with 22,145 observations from 3,252 companies, accounting for 72.1% of the total number of listed company samples during the same period.

### Model design

The model analyzing the relationship between marketization and capital structure in this study is as follows:


$$LEN_{it}=\beta_{0}+\beta_{1}MKT_{it}^{2}+\beta_{2}MKT_{it}+\beta_{3}Control_{it}+\mu_{i}+\gamma_{t}+\varepsilon_{it}\;(model\;1)$$

In the model, *i* and *t* represent the company and year, respectively. *LENit* represents the capital structure of company *i* in year *t*, and *MKTit* represents the marketization level of the company in the same period. The variable *Controlit* is used to control for other factors that may influence capital structure, and *εit* represents the random disturbance term.

Leverage (*LEN*) is the ratio of total liabilities to total assets [30]. The marketization process variable (*MKT*) represents a series of social reforms, measured by the “Degree of Marketization Index” compiled by Fang Gang et al. [15].

Based on previous research by scholars [31–35], control variables include growth opportunities (*MB*), return on assets (*ROA*), company size (*SIZE*), shareholder dividends (*VDIV*), and fixed asset ratio (*FATR*), as well as state-owned equity participation (*CONTROLRESULT*) (He et al., 2022). Specific calculation methods are detailed in Table 1.

**Table 1:** Definitions of variables in *Model*1.

Variable	Definition
*LEN*	*(total debt)/ (the book value of assets)*
*MKT*	*marketization process: China’s marketization index by province*
*MB*	*The ratio of market price to book value*
*ROA*	*The annual rate of return on corporate assets*
*SIZE*	*The natural logarithm of the total turnover*
*VDIV*	*A dummy variable of shareholder dividends. If there is a dividend for the year, the variable is taken as 1, and vice versa as 0*
*FATR*	*(fixed assets)/(total assets)*

Gain a more accurate understanding of the relationship between capital structure and explanatory variables by including control variables in the analysis.

### Summary statistics

Table 2 presents descriptive statistics. The average capital structure of sample companies is 0.42, the median is 0.41, and the standard deviation is 0.21, indicating a generally reasonable capital structure among listed companies in China. The average marketization index is 9.65, the median is 9.86, and the standard deviation is 1.46. The book asset ratio, return on investment, dividend payout ratio, and fixed investment ratio for Chinese listed companies are 3.56, 0.04, 0.87, and 0.25, respectively. Table 3 shows the correlation matrix of variables, indicating the absence of multicollinearity issues among variables.

**Table 2:** Summary statistics and descriptive statistics of variables.

Variable	N	Mean	SD	p25	p50	p75	Min	Max
LEN	22145	0.420	0.210	0.250	0.410	0.580	0.0100	0.990
MKT	22145	9.650	1.460	8.860	9.860	10.76	5.060	12.39
MB	22145	3.560	4.010	1.660	2.620	4.260	0.110	307.02
ROA	22145	0.0400	0.120	0.0100	0.0400	0.0700	−0.300	0.370
SIZE	22145	21.63	1.530	20.57	21.45	22.53	16.33	28.64
VDIV	22145	0.870	0.340	1	1	1	0	1
FATR	22145	0.250	0.220	0.100	0.210	0.360	0	13.88

Note: *Excluding samples with missing control variables.

**Table 3:** Correlation coefficient matrix.

	LEN	MKT	MB	ROA	SIZE	VDIV	ASSET
LEN	1						
MKT	−0.052***	1					
MB	−0.034***	0.021***	1				
ROA	−0.194***	0.034***	0.002**	1			
SIZE	0.541***	0.0280***	−0.292***	0.028***	1		
VDIV	−0.090***	0.124***	−0.060***	0.016**	0.088***	1	
FATR	0.207***	0.002	−0.035***	−0.099***	0.180***	0.036***	1

Note: *** indicates significance at the 1% level, ** indicates significance at the 5% level, and * indicates significance at the 10% level.

## Section 4: Empirical results

### Baseline results

To investigate the effect of marketization on corporate capital structure, we estimate a series of linear panel data models. Specifically, we employ pooled OLS regressions, fixed-effects models with year and/or firm fixed effects, and regressions with clustered standard errors at the firm level. The inclusion of year and firm fixed effects helps control for time-invariant firm-specific characteristics and macroeconomic shocks across years. We also use firm-clustered standard errors to correct for heteroskedasticity and within-firm correlation.

Table 4 presents the baseline regression results examining the effect of the marketization process on corporate capital structure. Column (1) reports the results from a pooled OLS regression without any fixed effects. Column (2) controls for year fixed effects. Column (3) further adds a set of control variables including MB, ROA, SIZE, VDIV, and FATR. Column (4) introduces both firm-level and year fixed effects to account for time-invariant firm characteristics and common temporal shocks. Column (5) reports results using clustered standard errors at the firm level to address heteroskedasticity and within-firm correlation. The coefficient of the quadratic term of the marketization process (MKT2) is significantly positive across all specifications, while the linear term (MKT) is significantly negative. These results consistently support the existence of a U-shaped relationship between marketization and corporate capital structure.

**Table 4:** Regression analysis of the marketization process on corporate capital structure.

Len	(1)	(2)	(3)	(4)	(5)
MKT2	0.0030***	0.0033***	0.0020***	0.0025***	0.0025***
	(−0.001)	0	0	0	(−0.001)
MKT	−0.0619***	−0.0408***	−0.0450***	−0.0453***	−0.0453***
	(−0.009)	(−0.008)	(−0.007)	(−0.007)	(−0.013)
MB			0.0048***	0.0055***	0.0055***
			0	0	(−0.001)
ROA			−0.1112***	−0.1119***	−0.1119
			(−0.006)	(−0.006)	(−0.112)
SIZE			0.0605***	0.0635***	0.0635***
			(−0.001)	(−0.001)	(−0.005)
VDIV			−0.0109***	−0.0076***	−0.0076
			(−0.002)	(−0.002)	(−0.005)
FATR			0.0699***	0.0923***	0.0923***
			(−0.004)	(−0.004)	(−0.027)
Constant	0.7351***	0.4987***	−0.6614***	−0.7694***	−0.7694***
	(−0.041)	(−0.037)	(−0.043)	(−0.048)	(−0.126)
YearFE	No	No	No	Yes	Yes
FirmFE	No	Yes	Yes	Yes	Yes
Cluster	No	No	No	No	Yes
Adj-R2	0.0042	−0.1426	0.0206	0.0375	0.1789
N	22145	22145	22145	22145	22145
Firm Observed number	3252	3252	3252	3252	3252

Note: *p < 0.10, **p < 0.05, ***p < 0.01

### Moderation analysis

In Table 5, Model (1) shows that the interaction term of state ownership and the quadratic term of marketization process (MKT2CONTROLRESULT) has a significantly negative coefficient (−0.0014, p < 0.01), and the interaction term with the linear term of marketization process (MKTCONTROLRESULT) has a significantly positive coefficient (0.0190, p < 0.01), indicating that state ownership has a negative moderating effect on the relationship between marketization process and corporate capital structure. Hypothesis H2 is supported.

**Table 5:** Results of moderation analysis.

LEN	(1)	(2)
MKT2	0.0022***	0.0010**
	(0)	(0)
MKT2CONTROLRESULT	−0.0014***	
	(0)	
MKT2FATR		0.0015***
		(0)
MKTCONTROLRESULT	0.0190***	
	(−0.002)	
MKTFATR		−0.0018
		(−0.005)
MKT	−0.0385***	−0.0295***
	(−0.008)	(−0.008)
MB	0.0078***	0.0075***
	(−0.002)	(−0.002)
ROA	−0.361	−0.3566
	(−0.256)	(−0.251)
SIZE	0.0771***	0.0814***
	(−0.002)	(−0.002)
VDIV	−0.0695***	−0.0748***
	(−0.004)	(−0.004)
Constant	−1.0376***	−1.1029***
	(−0.053)	(−0.052)
Year control	Y	Y
Adj-R2	0.3894	0.3885
N	22145	22145

Note: * p < 0.10, ** p < 0.05, *** p < 0.01

In Model (2) of Table 5, the interaction term of fixed asset ratio and the quadratic term of the marketization process (MKT2FATR) has a significantly positive coefficient (0.0015, p < 0.01), while the interaction term with the linear term of marketization process (MKTFATR) is not significant. This suggests that the fixed asset ratio has a positive moderating effect on the impact of the marketization process on corporate capital structure. Hypothesis H3 is supported.

### Robustness checks

(1) **Propensity Score Matching (PSM)**

To address potential sample selection bias arising from systematic differences between firms at different levels of marketization, we employ the Propensity Score Matching (PSM) method. This technique allows us to construct a matched sample of treatment and control groups with comparable observable characteristics, thereby enhancing the credibility of causal inference.

The balance test results in Table 6 show that there were significant differences at the 1% level in the means of variables (MB, ROA, SIZE, VDIV) between the treatment and control groups before matching. After matching, no significant differences are observed, indicating that propensity score matching satisfies the balance requirements. Table 7 presents the regression analysis on the matched data, where the quadratic term of the marketization process is significantly positive, and the linear term is significantly negative, with p-values all within the 1% significance range. The results are consistent with the empirical results before PSM. Hypothesis H1 is supported.

**Table 6:** PSM balance test results.

Variable	Unmatched	Mean		t-test	
	Matched	Treated	Control	t	p > |t|
MB	U	3.671	3.421	4.610	0
	M	3.6464	3.7373	−1.67	0.095
ROA	U	.04017	.02908	6.96	0
	M	.04011	.03925	1.08	0.278
SIZE	U	21.548	21.733	−8.97	0
	M	21.548	21.548	−0.50	0.358
VDIV	U	.88988	.84064	10.78	0
	M	.88984	.89136	−0.39	0.7
FATR	U	.23844	.2754	−12.50	0
	M	.23843	.23762	0.34	0.732

**Table 7:** Regression analysis of the marketization process on corporate capital structure with PSM.

LEN	Coefficient	Robust std. Err.	t	P > |t|	[95% conf. interval]	
MKT2	0.0013	0.0004	2.8300	0.0050	0.0004	0.0021
MKT	−0.0255	0.0082	−3.1200	0.0020	−0.0415	−0.0095
MB	0.0105	0.0004	29.8400	0.0000	0.0098	0.0112
ROA	−1.2693	0.0177	−71.6300	0.0000	−1.3040	−1.2346
SIZE	0.0890	0.0008	111.3300	0.0000	0.0874	0.0905
VDIV	−0.0666	0.0035	−19.1200	0.0000	−0.0734	−0.0598
FATR	0.0634	0.0071	8.9500	0.0000	0.0495	0.0772
_cons	−1.2894	0.0407	−31.7100	0.0000	−1.3691	−1.2097
YearFE	Y					
FirmFE	Y					
Adj-R2	0.4939

(2) **Instrumental variables**

To address potential endogeneity issues—such as reverse causality between marketization and capital structure, or omitted variable bias—we adopt an instrumental variable (IV) approach. Following Ma et al. (2022) and related literature [36], we use the firm’s registered location (i.e., province) as an instrumental variable, given that marketization levels vary substantially across provinces and evolve independently of individual firm decisions.

The underlying rationale is that while a firm’s location strongly influences the institutional environment it operates within (e.g., legal systems, market openness, government intervention), it is unlikely to be directly correlated with the firm’s idiosyncratic capital structure decisions, thus satisfying the relevance and exclusion conditions of a valid instrument.

In columns (2) and (3) of Table 8, we include the location dummy variable (Eeprovinc) as the instrumental variable for the marketization index. The Hausman test (p-value = 0) rejects the null hypothesis of exogeneity, confirming that endogeneity is a concern in the baseline model. The overidentification test (p-value = 1) suggests that the instrument is not correlated with the model’s error term. Moreover, the first-stage regression yields an F-statistic of 2053, far exceeding the conventional threshold of 10, which rules out the presence of weak instruments.

**Table 8:** Instrumental variables.

LEN	ols	first	iv
	(1)	(2)	(3)
MKT2	0.0025***		−0.0003***
	−0.001		0
MKT	−0.0453***		
	−0.013		
MB	0.0055***	0.1073***	0.0075***
	−0.001	0.031	0
ROA	−0.1119	3.5317***	−0.3590***
	−0.112	0.9910	−0.01
SIZE	0.0635***	0.6611***	0.0816***
	−0.005	0.0818	−0.001
VDIV	−0.0076	3.5724***	−0.0752***
	−0.005	0.3487	−0.003
FATR	0.0923***	−10.4469	0.1130***
	−0.027	0.6122	−0.006
eeprovice		36.09745***	
		(0.2382)	
_cons	−0.7694***	43.5639***	−1.4289***
	−0.126	(1.8247)	(0.0431)
Adj-R2	0.1789	0.5817	0.3868
N	22145	22,145	22,145
Hausman p-value			0
The P-value of Overid Test			1

note: *p < 0.10, **p < 0.05, ***p < 0.01

These test results support the validity of our instrumental variable strategy and reinforce the robustness of the main findings. Hypothesis H1—that marketization and capital structure exhibit a U-shaped relationship—is therefore supported.

(3) **Utest test method**

For the examination of the curvilinear relationship, Lind and Mehlum (2010) developed the Utest testing method based on Sasabuschi (1980) [37]. The null hypothesis stipulates an increase (or decrease) on one side of the interval and a decrease (or increase) on the other side, with the extreme point falling within the confidence interval of the independent variable. The results in Table 9 indicate that, before the extreme point, there is a negative correlation between the marketization process and corporate capital structure (-0.02, p = 0.00172). However, after reaching the extreme point, the correlation turns positive (0.0165, p = 0.00005). The estimated turning point of the U-shaped curve is 9.08347 on the marketization index scale, with a 95% confidence interval of (7.473, 10.116), indicating the nonlinear effect is statistically robust. Given that the average marketization score in the sample is approximately 8.6, this turning point lies within a realistic and policy-relevant range.The extreme point in the test results is 9.08347, with a 95% confidence interval range of (7.473, 10.116). Hypothesis H1 is supported.

**Table 9:** Utest test results.

U-test	Lower bound	Upper bound	Overall
Interval	5.061	12.39	
Slope	−0.0200	0.0165	
t-value	−2.928	3.908	2.93
P > |t|	0.00172	0.00005	0.00172
Extreme point			9.08347

This finding suggests that in less marketized regions (below the threshold), firms tend to deleverage due to institutional constraints and limited access to financing. In contrast, once the marketization level surpasses the turning point, more developed market mechanisms enhance firms’ ability and incentives to increase leverage. Given that the average marketization index is approximately 8.6, most regions lie near the critical point. This indicates that market-oriented reforms can significantly improve capital access, facilitating a shift from conservative to more proactive financing strategies. As marketization progresses, firms’ leverage behavior changes systematically, and the marginal impact of policy interventions may also evolve. Overall, the results underscore the economic significance of the U-shaped relationship and provide practical insights for shaping market reform and financial policy in transitional economies.

## Section 5: Conclusion

This study provides new empirical evidence on the relationship between corporate capital structure and the marketization process, particularly within the context of China’s transitional economy. It confirms a U-shaped relationship, where leverage initially decreases and then increases with the progression of marketization. Moreover, state ownership serves as a negative moderator, weakening this relationship, while the proportion of fixed assets acts as a positive moderator, strengthening it.

These findings imply that state-owned enterprises (SOEs), often criticized for inefficiency, may actually function as financial stabilizers by leveraging policy-based financing support during institutional transitions [17]. The U-shaped pattern also highlights the segmented nature of institutional development in emerging markets and underscores the importance of sequencing financial and legal reforms [29,38]. Labor regulation and enforcement, in particular, have differentiated impacts across ownership types and levels of marketization [20].

For policymakers, the findings recommend two-tiered strategies: promoting market-oriented reforms and fair competition in less-developed regions, while strengthening oversight and preventing monopolistic behavior in more advanced economies. Additionally, asset structure and ownership form should be recognized as tools for guiding capital formation and financial stability [1,27]. From a managerial perspective, firms should account for the marketization level when making capital structure or acquisition decisions. In lower-marketization contexts, firms tend to deleverage, whereas in highly marketized environments, leverage rises alongside improved access to financing.

Finally, these insights are relevant for other emerging and developing countries, particularly those with hybrid ownership systems or gradual privatization efforts. China’s case illustrates the importance of aligning institutional development with financial liberalization [29]. This study has some limitations. Due to data constraints, industry-specific factors such as competition intensity and firm age were not included, which may affect the model’s explanatory power. Future research could incorporate these variables, apply mixed-method approaches, or conduct cross-country comparisons to assess the generalizability of the U-shaped relationship between marketization and leverage. Overall, the findings offer valuable insights for China and other emerging economies undergoing institutional transitions.
